# Abdominoplasty Improves Quality of Life, Psychological Distress, and Eating Disorder Symptoms: A Prospective Study

**DOI:** 10.1155/2014/197232

**Published:** 2014-11-24

**Authors:** Kai M. M. Saariniemi, Asko M. Salmi, Hilkka H. Peltoniemi, Marjo H. Helle, Pia Charpentier, Hannu O. M. Kuokkanen

**Affiliations:** ^1^Department of Plastic and General Surgery, Vaasa Central Hospital, Hietalahdenkatu 2-4, 65130 Vaasa, Finland; ^2^Plastic Surgery Clinic KL, Uudenmaankatu 38, 00120 Helsinki, Finland; ^3^Department of Plastic Surgery, Tampere University Hospital, P.O. Box 2000, 33521 Tampere, Finland; ^4^Center for Eating Disorders, Fredrikinkatu 20 A 10, 00120 Helsinki, Finland

## Abstract

*Background*. Only some studies provide sufficient data regarding the effects of nonpostbariatric (aesthetic) abdominoplasty on various aspects of quality of life. Nevertheless, when considering the effects on eating habits, publications are lacking. Therefore we decided to assess the effects of nonpostbariatric abdominoplasty on eating disorder symptoms, psychological distress, and quality of life. *Materials and Methods*. 64 consecutive women underwent nonpostbariatric abdominoplasty. Three outcome measures were completed: the Eating Disorder Inventory (EDI), Raitasalo's modification of the Beck Depression Inventory (RBDI), and the 15D general quality of life questionnaire. *Results*. The mean age at baseline was 42 years and the mean body mass index (BMI) 26.4. Fifty-three (83%) women completed all the outcome measures with a mean follow-up time of 5 months. A significant improvement from baseline to follow-up was noted in women's overall quality of life, body satisfaction, effectiveness, sexual functioning, and self-esteem. The women were significantly less depressive and had significantly less drive for thinness as well as bulimia, and their overall risk of developing an eating disorder also decreased significantly. *Conclusions*. Abdominoplasty results in significantly improved quality of life, body satisfaction, effectiveness, sexual functioning, self-esteem, and mental health. The risk of developing an eating disorder is decreased significantly. This trial is registered with Clinicaltrials.gov NCT02151799.

## 1. Introduction

Only few studies provide some sufficient data regarding the effects of nonpostbariatric (aesthetic) abdominoplasty on various aspects of quality of life [1–4]. In these studies, an improvement in body image, self-esteem, mental health, sexual relations, functioning and satisfaction, and quality of life has been observed. However, the level of evidence is considered weak [5]. When regarding the effects on eating habits, publications are lacking. In addition, the recent case in European Court Justice, looking whether plastic or cosmetic surgery is subject to VAT or not, raised the need to study the effects of such procedures on related quality of life aspects [6]. Sometimes reconstructive aesthetic surgery can also have considerable health-improving effects and therefore be VAT-exempt. Therefore we decided to assess the effects of nonpostbariatric abdominoplasty on eating disorder symptoms, psychological distress, and quality of life. With validated questionnaires, comparison to other health conditions is enabled, and, consequently, the health effect is put into perspective.

## 2. Materials and Methods

This study consists of 64 consecutive women who underwent nonpostbariatric abdominoplasty at the Plastic Surgery Clinic KL, Helsinki, Finland. The Surgical Ethics Research Committee of the Pirkanmaa Hospital District provided ethical approval (registration number R09166). The women completed three outcome measures at baseline and at follow-up: the Eating Disorder Inventory (EDI), Raitasalo's modification of the Beck Depression Inventory (RBDI), and the 15D general quality of life questionnaire. Demographic data was obtained by an interview and a preliminary information form. Possible complications such as hematoma, seroma, infection, wound healing problems, or scar hypertrophy were recorded at the follow-up.

The women were operated on by one plastic surgeon (A.S.). Preoperative markings were made in the standing position. Patients underwent conventional abdominoplasty combined with preceding lidocaine-adrenaline-saline-infiltration and progressive tension suture closure-technique. Complementary liposuction was performed when needed as well as rectus muscle plication or umbilical hernia repair. All operations were done under general anaesthesia. A prophylactic antibiotic of 1.5 g of cefuroxime was intravenously administered preoperatively and 20–40 mg of enoxaparin subcutaneously at the end of the operation. No drains were used. Patients wore an elastic belt for four weeks and avoided heavy lifts (>10 kg) for four to five weeks. Discharge was planned the next day.

### 2.1. Outcome Measures

The Eating Disorder Inventory (EDI) is a diagnostic tool designed for use in a clinical setting to assess the presence of an eating disorder [7]. This self-report questionnaire comprises 64 questions divided into eight subscales (drive for thinness, bulimia, body dissatisfaction, ineffectiveness, perfectionism, interpersonal distrust, interoceptive awareness, and maturity fears). Threshold values are used when assessing clinical relevancy (Charpentier P., Finnish version of the Eating Disorder Inventory, unpublished data 2001).

The RBDI mood questionnaire [8] is Raitasalo's modification of the short form of the Beck Depression Inventory (BDI) [9, 10], and it has been used in Finland for nearly 30 years.

General health-related quality of life (HRQoL) was measured by the 15D. It is a generic, 15-dimensional, standardized, self-administered HRQoL instrument that can be used both as a profile and as a single index score measure [11].

### 2.2. Statistical Analysis

The data were analyzed with the aid of the PASW Statistics 18.0 for Macintosh. The algorithm for the basic scoring of 15D ran on PASW was obtained from the developer of the instrument. Missing values for the Eating Disorder Inventory questionnaire were replaced by the Missing Value Analysis estimation method of PASW (median values of two nearby points). At baseline 23 values (0.56%) and none at follow-up were missing for the Eating Disorder Inventory (EDI) questionnaire and were replaced by the PASW. At most, three out of 64 answers were replaced for one case.

Data is expressed as mean (standard deviation, SD) or frequency (percentage). From baseline to follow-up, normally distributed data were compared with the paired *t*-test, and the Wilcoxon signed rank test was applied for skewed or categorical data. The anxiety and depression categories were dichotomized into “symptomatic” and “non-symptomatic.” Changes from baseline to follow-up for dichotomized data were tested with the McNemar test. Probabilities of less than 0.01 were considered significant. A comparison of the patients' quality of life with the age-standardized general population [12] was performed with the Mann-Whitney *U* test. Probabilities of less than 0.05 were considered significant.

## 3. Results

The mean age at baseline was 42 years (SD 10.2). Mean height and weight were 165 cm (SD 6.4) and 72 kg (13.8), respectively. The mean body mass index (BMI) was 26.4 (SD 4.3). Eighteen (28%) women reported having comorbidities (five with hypothyreosis, three with asthma, three with hypertension, three with diabetes, one with depression, one with celiac disease, one with multiple sclerosis, and one with systemic lupus erythematosus).

Mean resection weight was 1478 grams (SD 1023). Twenty-five (39%) women had complementary liposuction with a mean volume of 567 mL (SD 209). All women had rectus plication. Eight (13%) women had concomitant umbilical hernia repair (1-2 cm in diameter). Two women stayed in the hospital for two days; all others were discharged the next day after the operation. One woman required a reoperation due to a hematoma. Blood transfusion was not needed. Eight (13%) women had a superficial knot fistulation/infection and three (5%) wound dehiscence. All resolved with antibiotics and local wound care. Thus the overall major complication rate was 2% and the minor complication rate 17%.

All women had at least one postoperative visit with a mean follow-up time of 4 months (SD 2.9). Fifty-three (83%) women of these completed all the outcome measures with a mean follow-up time of 5 months (SD 2.7). Women who did not fill out the questionnaires did not differ in their baseline characteristics when compared to those who did (data not shown).

Significantly less drive for thinness as well as bulimia could be observed postoperatively (Table 1). Body satisfaction and effectiveness were improved, and the overall risk of an eating disorder was significantly reduced. Of the 53 women analyzed, seven (13%) preoperatively had EDI summary scores comparable to clinical cases. Postoperatively only one woman (2%) had such EDI scores. This change was statistically significant (*P* = 0.016). Self-esteem and depression improved from baseline to follow-up (Table 1).

Overall quality of life (15D index score, *P* = 0.004) as well as the dimension sex (*P* = 0.045) improved significantly after the operation. At baseline, the women in the study population had a worse quality of life in the dimension sleep when compared to the age-standardized general population (*P* > 0.05). At follow-up, the difference was not statistically significant although the women in the study population still had inferior values. However, the dimensions of discomfort and symptoms (*P* > 0.001), depression (*P* > 0.001), distress (*P* > 0.05), and vitality (*P* > 0.05) demonstrated superior values when compared to the age-standardized general population.

## 4. Discussion

We found in our prospective study that nonpostbariatric abdominoplasty significantly improves women's overall quality of life, body satisfaction, effectiveness, sexual functioning, and self-esteem. The women were also significantly less depressive. Similar effects have been noted in previous studies [1–4]. However, as far as we know, our findings that less eating disorder symptoms are noted after abdominoplasty have not been presented before. We found that women had significantly less drive for thinness as well as bulimia. Their overall risk of developing an eating disorder also decreased significantly. In addition, there were postoperatively significantly fewer women having scores comparable to clinical cases.

The women scored postoperatively significantly better values in general quality of life. In addition, the dimension sex improved significantly. The latter has also been noted by others [1, 2, 4]. This is a natural consequence of abdominoplasty as the abdominal area plays an important role in psychosexual functioning. However, an overall improvement in quality of life (demonstrated by a nonspecific, less sensitive general instrument) has not been demonstrated previously, and this underlines the total impact that abdominoplasty has on quality of life.

However, at the baseline the women included in the study population reported significantly worse quality of sleep when compared to the general female population. This difference decreased to nonsignificant at follow-up, but the values of the study population were still inferior. This may reflect some preoperative psychological distress that is not related to concerns in the abdominal area and therefore not resolved by abdominoplasty. However, postoperatively the study population scored significantly better in the dimensions discomfort and symptoms, depression, distress, and vitality when compared to the general population. This, on the other hand, reflects the detailed effects abdominoplasty has on quality of life.

Preoperatively only one woman self-reported a depressive disorder. However, according to the mood questionnaire, six women were found to be depressive and/or anxious. As excess psychological distress may negatively affect outcome [13, 14], our findings support routine, validated assessment of preoperative psychological distress.

There are some limitations to our study. The mean follow-up time was five months, but 20 (31%) women had a follow-up time of less than two months. Therefore, the findings in our study may change over time [2]. Therefore studies with a longer follow-up are warranted. This is our plan in the near future.

Eight (13%) women preoperatively had EDI summary scores comparable to clinical cases. This is higher than in population based studies where life time prevalence has been found to be roughly at 1–4%, 1–3%, and 3% for anorexia nervosa, bulimia nervosa, and eating disorders otherwise specified, respectively [15]. A screening rather than a comprehensive interview approach was taken in assessing eating disorder symptoms to ease compliance. However, questionnaire-derived information alone cannot be used to arrive at a diagnosis of psychopathology. Therefore no final conclusions can be drawn from this study regarding the prevalence of eating disorders among abdominoplasty patient populations.

The recent case in European Court Justice, looking whether plastic or cosmetic surgery is subject to VAT or not, raised the need to study the effects of such procedures on related quality of life aspects. Our study demonstrates that procedures traditionally classified as aesthetic have also a significant impact on quality of life. Therefore the VAT exemption has to be considered also for these procedures.

## 5. Conclusions

Nonpostbariatric (aesthetic) abdominoplasty significantly improves women's overall quality of life, body satisfaction, effectiveness, sexual functioning, self-esteem, and mental health. Less drive for thinness as well as bulimia is noted and the overall risk of developing an eating disorder is decreased significantly. However, to confirm and strengthen our results, further studies with longer follow-up are needed.

## Figures and Tables

**Table 1:** Values for the Eating Disorder Inventory (EDI) and Raitasalo's modification of the Beck Depression Inventory (RBDI) for women having nonpostbariatric (aesthetic) abdominoplasty (*N* = 53).

	Baseline score	Follow-up score	*P* value
Drive for thinness	4.81 (5.16)	2.96 (3.84)	<0.001
Bulimia	0.73 (1.61)	0.25 (0.81)	0.016
Body dissatisfaction	9.08 (4.83)	4.26 (2.95)	<0.001
Ineffectiveness	0.70 (1.59)	0.28 (0.74)	0.048
Perfectionism	2.40 (3.23)	2.23 (2.79)	0.951
Interpersonal distrust	0.61 (1.50)	0.47 (1.08)	0.326
Interoceptive awareness	1.20 (2.10)	0.74 (1.44)	0.170
Maturity fears	2.16 (1.93)	2.25 (1.65)	0.850
EDI summary	21.69 (15.10)	13.43 (9.91)	<0.001

Depression score	1.81 (2.18)	0.74 (1.35)	<0.001
Self-esteem score	7.13 (3.13)	8.64 (3.20)	<0.001

Anxious	6 (11)	3 (6)	0.289
Depressive	6 (11)	1 (2)	0.031

Values are mean (SD) for scores and frequencies (%) for cases. Wilcoxon signed rank test for scores, McNemar test for cases.
